# Navigating Recovery After Medical Adverse Events: The Role of Social Support and Cognitive Reappraisal in Second Victims’ Post‐Traumatic Growth

**DOI:** 10.1155/jonm/5142544

**Published:** 2026-07-13

**Authors:** Yanfang Long, Lin Zhuo, Renfang Liang, Yuting Zeng, Li Li

**Affiliations:** ^1^ Teaching and Research Section of Clinical Nursing, Xiangya Hospital, Central South University, Changsha, Hunan, China, csu.edu.cn; ^2^ Department of General Medicine, Xiangya Hospital, Central South University, Changsha, Hunan, China, csu.edu.cn; ^3^ Central South University Xiangya School of Nursing, Changsha, Hunan, China

**Keywords:** cognitive reappraisal, emotion regulation, medical adverse event, post-traumatic growth, second victim, social support

## Abstract

**Background:**

Healthcare providers involved in a medical adverse event, referred to as second victims, frequently suffer negative psychological distress. However, little was known about the post‐traumatic growth of this population from a positive perspective.

**Objective:**

To examine the level of post‐traumatic growth among second victims of medical adverse events and to explore the factors associated with this growth.

**Design:**

A multisite, cross‐sectional study.

**Setting:**

Four tertiary hospitals in Changsha city, Hunan province, China.

**Participants:**

A final sample of 237 medical staff who had been exposed to and reported at least one prior adverse event previously participated in the survey from July to October 2022.

**Methods:**

Utilizing a two‐stage stratified random sampling technique, participants were recruited to complete an online survey measuring sociodemographic characteristics, post‐traumatic growth, perceived social support, and emotion regulation strategies. Multivariable linear regression analysis was conducted to identify significant correlates of post‐traumatic growth.

**Results:**

Of the 237 valid respondents, 71.31% were nurses and 40.08% experienced a level III adverse event (resulting in no patient harm). The average total score for post‐traumatic growth was 2.53 ± 1.26. The mean scores for each dimension were 2.66 ± 1.31 for personal strength, 2.66 ± 1.22 for appreciation of life, 2.54 ± 1.22 for relating to others, 2.43 ± 1.24 for new possibilities, and 2.32 ± 1.28 for spiritual change. Support from significant others, cognitive reappraisal, workplace environment, less severe adverse event, younger age, shorter length of employment, and an introverted personality type were significantly associated with post‐traumatic growth.

**Conclusion:**

Second victims of medical adverse events demonstrated low‐to‐moderate levels of post‐traumatic growth. Perceived support from significant others and the use of cognitive reappraisal strategies are key factors associated with positive psychological changes among health care providers following an adverse event.

**Implications for Nurse Managers:**

Nurse managers should prioritize hospital‐based, institutional support mechanisms rather than relying solely on second victims’ personal relationships. This includes implementing structured peer‐support systems, conducting formal debriefing, and cultivating a nonpunitive organizational culture of psychological safety to foster post‐traumatic growth following adverse clinical events.

**Summary**
• What is already known?
◦ Positive changes have been widely reported by survivors of various traumatic events, such as traffic accidents, natural disasters, interpersonal trauma, and medical diagnoses, manifesting in improved relations with others, the identification of new possibilities, increased personal strength, spiritual change, and an enhanced appreciation of life.
◦ Medical adverse events are highly stressful for the affected healthcare professionals. While negative emotional reactions are well‐documented, the potential for positive adjustment or growth has remained underexplored.
◦ The post‐traumatic affective–cognitive processing model establishes a conceptual framework to examine the variables associated with the development and consequences of post‐traumatic growth.
• What this paper adds?
◦ This study evaluates the positive psychological outcomes of healthcare professionals involved in medical adverse events, revealing a low‐to‐moderate baseline of post‐traumatic growth.
◦ Support from significant others, cognitive reappraisal, workplace environment, event severity, age, length of employment, and introverted personality traits were identified as significant factors associated with post‐traumatic growth in affected healthcare professionals.
◦ Enhanced support from significant others (such as leaders and colleagues) is linked to lower perceived stress following adverse events. Consequently, clinical nursing managers and researchers should focus on developing cognitive reappraisal strategies and building highly supportive workplace environments.

## 1. Introduction

Ensuring patient safety remains a fundamental priority for hospitals and healthcare providers (HCPs). However, since “to err is human” [1], adverse events (AEs), defined as injuries caused by medical management rather than an underlying disease [1, 2], are inevitable in clinical practices. A scoping review showed that approximately 10% of hospitalized patients are affected by at least one AE globally, with 7.3% of these events resulting in fatal outcomes [3].

Patients and their families, who suffer directly from the AE, are recognized as the primary victims [4]. Meanwhile, HCPs involved in the same incident—termed “second victims” by Wu [4]—also warrant considerable attention. They often experience feelings of guilt, shame, and fear [4–6], accompanied by acute stress reactions, such as shock, anxiety, depressive symptoms, social withdrawal, and agitation [6–8]. These negative emotions, if not managed appropriately, can place exposed HCPs at long‐term risk for burnout and post‐traumatic stress disorders [9, 10]. It may take traumatized HCPs weeks or even years to fully recover from this emotional distress, depending on the nature of the incident and the severity of patient harm [9].

Apart from these negative psychological challenges, the emotional impact of AEs may also offer opportunities for positive psychological changes [11], known as post‐traumatic growth (PTG). PTG manifests as improved relations with others, the identification of new life possibilities, increased personal strength, spiritual change, and an enhanced appreciation of life [12]. This growth does not occur as a direct result of the trauma itself; instead, it arises from the psychological struggle to process and accept the new reality [13]. A large number of studies have explored the PTG phenomenon since its conceptual introduction [14–16]. However, nearly all prior work has focused on populations undergoing challenging life circumstances, such as cancer, physical accidents, natural disasters, or bereavement. To our knowledge, there is a lack of empirical research regarding PTG levels among second victims in the aftermath of clinical AEs.

Based on qualitative interviews with 31 healthcare professionals, a six‐stage recovery trajectory was identified to describe the second victim phenomenon: chaos and accident response, intrusive reflections, restoring personal integrity, enduring the inquisition, obtaining emotional first aid, and moving on. In the final stage, second victims generally follow one of three paths: dropping out, surviving, or thriving. By thriving, HCPs demonstrate a positive legacy, reporting that they learned constructive lessons, adapted their clinical behaviors, and actively engaged in subsequent quality improvement processes [17].

This recovery framework demonstrates that HCPs share similar emotional reactions following AE exposure and continually regulate their emotions throughout the process. Emotion regulation refers to “the processes by which individuals influence which emotions they have, when they have them, and how they experience and express them” [18]. The most widely studied emotion regulation strategies are cognitive reappraisal and expressive suppression. Through reappraisal, affected HCPs alter how they construe an AE before stress‐related emotions arise, whereas expressive suppression occurs after emotional responses are generated by inhibiting their outward expression [19]. Beyond internal cognitive processing, social support is a key factor in fostering PTG. Higher perceived social support has been associated with elevated levels of PTG in chronic disease populations [20]. However, limited research has evaluated the concurrent roles of emotion regulation and social support on the PTG of HCPs involved in AEs.

Given the dual importance of maintaining patient safety and protecting HCPs’ psychological well‐being, investigating PTG in this group is essential to fostering positive outcomes. Currently, there is a paucity of studies evaluating positive adaptations among HCPs exposed to AEs. Furthermore, it is necessary to identify the variables associated with this growth. According to the post‐traumatic affective–cognitive processing model, individuals can achieve growth‐oriented outcomes driven by intrinsic motivations, and the cognitive appraisal process is heavily shaped by personality, coping, and social support factors [21]. Nonetheless, this model has not yet been applied to HCPs who have been exposed to medical errors. Thus, this study evaluates the positive psychological legacy of HCPs exposed to AEs and identifies the potential correlates, establishing a foundation for supportive interventions. Based on the model, the following hypotheses were evaluated:

 H1: Cognitive reappraisal is positively associated with PTG.
 H2: Higher perceived social support (from family, friends, and significant others) is positively associated with PTG.
 H3: Together with other covariates, cognitive reappraisal and social support jointly explain a significant proportion of the variance in PTG.

## 2. Methods

### 2.1. Study Design and Setting

A multicenter, cross‐sectional survey was conducted across four tertiary hospitals to evaluate HCPs’ PTG following exposure to AEs from July to October 2022. Reporting adheres to the Strengthening the Reporting of Observational Studies in Epidemiology (STROBE) guidelines. The SH9 classification of medical AEs developed by Wei & Tian [22] was utilized. This system categorizes AEs into four main levels based on their impact on patients, with level I (warning event) being the most severe and level IV (near miss) the least severe.

Four tertiary hospitals in Changsha city were selected via a two‐stage stratified sampling method. First, four of the six administrative districts (Yuelu, Kaifu, Yuhua, Tianxin, Wangcheng, and Furong) in Changsha were randomly selected. Subsequently, one tertiary hospital was randomly selected from each participating district.

### 2.2. Participants

HCPs with documented AE exposure and reporting experiences from the four selected hospitals were invited to participate. Eligibility criteria required participants to be front‐line physicians, nurses, or technicians who were primarily responsible for at least one AE that occurred between January 2021 and June 2022 and who reported experiencing emotional distress as a result of the incident. HCPs were excluded if they had experienced a major confounding life crisis (e.g., bereavement of an immediate family member or a personal life‐threatening illness) within the past 3 years, or if they were on extended leave during the active data collection window.

A pilot survey was conducted among 30 eligible HCPs, yielding an average PTG item score of 3.06 (*μ*) with a standard deviation of 1.11 (*δ*). Based on these pilot findings, the sample size was calculated to be 202 according to the formula *n* = ((*Z* *α*∗*δ*)/(*ε*∗*μ*))^2^, with a two‐sided significance level *α* = 0.05 (*Z* = 1.96) and a relative allowable error *ε* = 0.05 [23]. Considering a potential 20% invalid response rate, the final sample size was adjusted to 242.

### 2.3. Measures

#### 2.3.1. Covariables: Sociodemographic Characteristics and AE Experiences

Sociodemographic variables collected included gender, age, marital status, education level, length of employment, practice specialty, professional title, annual family income, personality type, workplace environment, and family relationships. AE‐related variables included the total number of AEs experienced by the participant, the severity level of the most critical event, and whether the individual received formal institutional penalties.

#### 2.3.2. Dependent Variable: PTG

PTG was measured using the post‐traumatic growth inventory (PTGI) originally developed by Tedeschi and Calhoun [12] and adapted into Chinese by Wang et al. [24]. The scale consists of 20 items distributed across five domains: Relating to Others (6 items), New Possibilities (3 items), Personal Strength (4 items), Spiritual Change (3 items), and Appreciation of Life (4 items). Each item is rated on a 6‐point Likert scale ranging from 0 (I did not experience this change at all after the trauma) to 5 (I experienced this change very much after the trauma). Total scores range from 0 to 100, with higher scores indicating greater levels of positive growth. In this study, the PTGI demonstrated high internal consistency, with a Cronbach’s *α* of 0.930.

#### 2.3.3. Independent Variables: Emotion Regulation and Social Support

Emotion regulation strategies were assessed using the Emotion Regulation Questionnaire (ERQ) developed by Gross & John [25]. This 10‐item self‐report tool captures an individual’s tendency to manage emotional states through cognitive reappraisal (6 items) and expressive suppression (4 items). Each item is rated on a 7‐point Likert scale from 1 (strongly disagree) to 7 (strongly agree), with higher scores indicating more frequent deployment of that specific strategy. The ERQ in the current sample showed acceptable to good internal consistency, with a Cronbach’s *α* of 0.884 for cognitive reappraisal and 0.754 for expression suppression.

Participants’ perceived social support was evaluated using the 12‐item Perceived Social Support Scale (PSSS), translated and culturally adapted into Chinese by Jiang [26] based on the original Multidimensional Scale of Perceived Social Support (MSPSS) developed by Zimet et al. [27]. The scale measures perceived support across three distinct domains: family, friends, and significant others. Crucially, to justify its application in the workplace and ensure appropriate interpretation, items in the Chinese version’s “Other support” (Significant Others) subscale explicitly instruct respondents to evaluate support received from relatives, organizational leaders, and workplace colleagues. Each item is scored on a 7‐point Likert scale ranging from 1 (strongly disagree) to 7 (strongly agree). Cumulative scores range from 12 to 84, with higher values reflecting greater perceived social support. In the present study, the instrument demonstrated excellent internal consistency, with a Cronbach’s *α* of 0.934.

### 2.4. Data Collection

The data collection window spanned from July to October 2022. Following institutional authorization, the names of HCPs who were exposed to at least one AE between January 2021 and June 2022 were extracted from the respective hospital incident reporting systems. Staff entries were anonymized and assigned sequential numbers. Using a random number table method, 244 HCPs (61 from each hospital) were selected. Digital invitations containing a *Wenjuanxing*‐based online survey link were distributed to the selected sample. Completion required approximately 8–12 min, and the platform required all questions to be completed before submission.

### 2.5. Ethical Considerations

This study was approved by the Ethics Committee of Xiangya Hospital, Central South University (No. 2022020533), and the institutional boards of all participating hospitals. Informed consent was obtained from all participants digitally by requiring a mandatory “YES” response to a baseline screening prompt verifying voluntary participation after review of the study aims.

### 2.6. Data Analysis

Statistical analyses were executed using SPSS software (Version 20.0; IBM Corp.). Continuous variables with normal distributions are reported as mean ± standard deviation (M±SD); non‐normally distributed continuous variables are reported as medians and interquartile ranges. Categorical data are expressed as frequencies and percentages. Independent *t*‐tests or one‐way analyses of variance (ANOVAs) were used to compare PTG across distinct demographic groups. Pearson correlation analysis was used to examine correlations among perceived social support, emotion regulation, and the PTG score. Statistically significant variables in the univariate analyses were subsequently integrated into a multivariable linear regression model. All tests were two‐tailed, and *p* < 0.05 was considered statistically significant.

### 2.7. Validity, Reliability, and Rigor

To maintain high specificity regarding the second victim definition, respondents who selected “Not at all” to the question “How much were you emotionally affected after an AE exposure?” were excluded from the analysis, consistent with previous studies [10]. Platform‐level settings on *Wenjuanxing* prevented duplicate submissions from the same user ID and enforced complete item responses. Questionnaires were considered to be invalid and omitted during data cleaning if they exhibited uniform or repeating option selections throughout the survey, displayed an obvious systematic layout pattern, or failed a built‐in attention check question [28].

## 3. Results

A total of 244 questionnaires were collected, of which 7 were excluded as invalid, yielding a final sample of 237 valid questionnaires and an effective response rate of 97.13%.

### 3.1. Sample Characteristics

The detailed characteristics of the sample are displayed in Table 1. Of the 237 participants, the majority were female (74.68%), aged 30∼39 years (55.27%), married (55.70%), held a bachelor’s degree (52.74%), and practiced as nurses (71.31%). Nearly half of them (42.62%) have worked for 5–10 years; 31.22% rated their workplace environment as “good”, and 42.61% rated their family relationships as “good”. Regarding event exposure, 74.26% had experienced an AE only once. In terms of severity, 40.08% reported that their most critical event did not cause any patient harm (Level III), and 78.48% did not receive administrative penalties from their institution.

**TABLE 1:** General information of participants (*n* = 237).

Variable	Category	*N* (%)
Gender	Male	60 (25.32)
	Female	177 (74.68)

Age (years)	< 30	71 (29.96)
	30 ∼ 39	131 (55.27)
	40 ∼ 49	28 (11.82)
	≥ 50	7 (2.95)

Marital status	Unmarried	59 (24.90)
	Married	132 (55.70)
	Divorced	42 (17.72)
	Widowed	4 (1.68)

Education level	College	7 (2.95)
	Bachelor’s degree	125 (52.74)
	Master’s degree	81 (34.18)
	Doctorate	24 (10.13)

Length of employment (years)	< 1	8 (3.38)
	1 ∼ 2	16 (6.75)
	3 ∼ 4	56 (23.63)
	5 ∼ 9	101 (42.62)
	≥ 10	56 (23.63)

Practice specialty	Nurse	169 (71.31)
	Physician	57 (24.05)
	Technician	11 (4.64)

Professional title	None	14 (5.91)
	Primary	108 (45.57)
	Intermediate	96 (40.51)
	Senior	19 (8.01)

Annual family income (10,000 RMB)	< 15	51 (21.52)
	15 ∼ 29	147 (62.03)
	≥ 30	39 (16.45)

Personality type	Extroverted	97 (40.93)
	Introverted	54 (22.79)
	Intermediate	86 (36.28)

Workplace environment	Very good	43 (18.14)
	Good	74 (31.22)
	Average	54 (22.79)
	Poor	66 (27.85)

Family relationship	Very good	101 (42.61)
	Good	86 (36.29)
	Average	19 (8.02)
	Poor	31 (13.08)

Number of AEs experienced	Once	176 (74.26)
	Multiple times	61 (25.74)

Severity of the most severe event	Level I (warning event)	8 (3.38)
	Level II (with patient harm)	43 (18.14)
	Level III (no patient harm)	95 (40.08)
	Level IV (near miss)	91 (38.40)

Hospital punishment suffered	Yes	51 (21.52)
	No	186 (78.48)

### 3.2. PTG Scores

The overall mean PTG score was 2.53 ± 1.26. The mean scores for each dimension were 2.66 ± 1.31 for personal strength, 2.66 ± 1.22 for appreciation of life, 2.54 ± 1.22 for relating to others, 2.43 ± 1.24 for new possibilities, and 2.32 ± 1.28 for spiritual change. Table 2 presents the total and dimensional PTG scores. In terms of items, “Appreciating each day” (2.84 ± 1.32), “I accept needing others” (2.78 ± 1.16), and “Knowing I can handle difficulties” (2.75 ± 1.29) had the highest scores. Conversely, “I established a new path for my life” (2.16 ± 1.23), “I developed new interests” (2.21 ± 1.32), and “A sense of closeness with others” (2.32 ± 1.29) had the lowest scores.

**TABLE 2:** Total and dimensional scores of PTG (*n* = 237).

Dimension	Items	Score range	Composite score (M ± SD)	Mean score of items (M ± SD)
Overall PTG	20	0 ∼ 100	50.57 ± 16.33	2.53 ± 1.26
Self‐transformation	4	0 ∼ 20	9.30 ± 3.83	2.32 ± 1.28
Insights on life	6	0 ∼ 30	15.96 ± 5.44	2.66 ± 1.22
Relating to others	3	0 ∼ 15	7.62 ± 2.51	2.54 ± 1.22
New possibilities	4	0 ∼ 20	9.70 ± 3.86	2.43 ± 1.24
Personal strength	3	0 ∼ 15	7.99 ± 3.26	2.66 ± 1.31

### 3.3. Univariate Analysis of PTG

Group‐level evaluations revealed significant differences in PTG scores across age, marital status, education, length of employment, specialty, income, personality, workplace environment, family relationship, event frequency, and event severity (Table 3). Specifically, baseline scores were higher among individuals who were younger than 30 years old (*F* = 5.713, *p* = 0.001), were employed less than 1 year (*F* = 6.300, *p* < 0.001), worked as nurses (*F* = 9.791, *p* < 0.001), reported an extroverted personality (*F* = 4.633, *p* = 0.011), and experienced near‐miss AEs (*F* = 15.289, *p* < 0.001).

**TABLE 3:** Univariate analysis of PTG (*n* = 237).

Variable	Category	PTG score (M±SD)	t/F	*p*
Gender	Male	47.90 ± 19.45	2.165	0.143
	Female	51.48 ± 15.08		

Age (years)	< 30	53.18 ± 12.76	5.713	0.001
	30∼39	51.70 ± 15.39		
	40 ∼ 49	43.00 ± 23.00		
	≥ 50	33.43 ± 18.89		

Marital status	Unmarried	50.44 ± 18.69	3.353	0.020
	Married	52.30 ± 13.97		
	Divorced	44.24 ± 17.82		
	Widowed	62.00 ± 22.11		

Education level	College	60.86 ± 16.02	6.660	< 0.001
	Bachelor’s degree	46.91 ± 15.33		
	Master’s degree	52.46 ± 17.17		
	Doctorate	60.29 ± 12.68		

Length of employment (years)	< 1	66.38 ± 17.20	6.300	< 0.001
	1 ∼ 2	43.94 ± 17.98		
	3 ∼ 4	53.07 ± 17.29		
	5 ∼ 9	52.78 ± 13.75		
	≥ 10	43.73 ± 16.23		

Practice specialty	Nurse	53.30 ± 13.05	9.791	< 0.001
	Physician	42.63 ± 20.70		
	Technician	49.82 ± 23.41		

Professional title	None	51.50 ± 6.98	0.311	0.817
	Primary	51.57 ± 17.01		
	Intermediate	49.65 ± 17.29		
	Senior	48.95 ± 12.17		

Annual family income (10,000 RMB)	< 15	55.92 ± 12.81	3.749	0.025
	15 ∼ 29	49.48 ± 16.56		
	≥ 30	47.72 ± 18.31		

Personality type	Extroverted	53.70 ± 15.42	4.633	0.011
	Introverted	45.41 ± 18.30		
	Intermediate	50.29 ± 15.33		

Workplace environment	Very good	53.14 ± 18.50	7.116	< 0.001
	Good	55.23 ± 14.62		
	Average	50.91 ± 13.32		
	Poor	43.41 ± 16.76		

Family relationship	Very good	53.37 ± 12.74	13.613	< 0.001
	Good	53.21 ± 13.60		
	Average	50.00 ± 20.73		
	Poor	34.52 ± 21.49		

Number of AEs experienced	Once	52.55 ± 14.42	10.427	0.001
	Multiple times	44.87 ± 19.95		

Severity of the most severe event	Level I (warning event)	23.13 ± 26.29	15.289	< 0.001
	Level II (with patient harm)	42.74 ± 14.07		
	Level III (no patient harm)	53.22 ± 15.72		
	Level IV (near miss)	53.92 ± 13.42		

Hospital punishment suffered	Yes	46.88 ± 21.96	3.354	0.068
	No	51.59 ± 14.32		

### 3.4. Correlation Analysis of Perceived Social Support, Emotion Regulation, and PTG

Table 4 presents the bivariate correlations among perceived social support, emotion regulation, and PTG, as estimated using Spearman’s rank correlation. Perceived social support from significant others, family, and friends was all positively correlated with the PTG total score (*r* range: 0.520–0.538, *p* < 0.001). Regarding emotion regulation strategies, cognitive reappraisal showed a significant positive correlation with the PTG total score (*r* = 0.547, *p* < 0.001). In contrast, expressive suppression showed a significant negative correlation (*r* = −0.320, *p* < 0.001).

**TABLE 4:** Correlation analysis of perceived social support, emotional regulation, and PTG (*n* = 237).

Variable dimension	Items	M±SD	*r*	*p*
Perceived social support (total)	12	50.94 ± 14.06	0.590	< 0.001
Significant other	4	16.26 ± 5.56	0.538	< 0.001
Family	4	17.49 ± 5.37	0.520	< 0.001
Friends	4	17.19 ± 4.72	0.531	< 0.001
Emotional regulation (total)	10	42.25 ± 5.93	0.389	< 0.001
Cognitive reappraisal	6	26.21 ± 6.66	0.547	< 0.001
Expressive suppression	4	16.04 ± 4.17	−0.320	< 0.001

### 3.5. Multiple Linear Regression Analysis of Factors Associated With PTG

Table 5 presents the results of the multiple linear regression analysis exploring independent factors associated with PTG scores. The adjusted generalized variance inflation factor (GVIF) values for all independent variables ranged from 1.212 to 2.264, indicating no significant multicollinearity. Among all the variables included in the model, seven remained significant after controlling for all other factors, accounting for 50.70% of the total variance in second victims’ PTG. Specifically, higher perceived support from significant others (*B* = 0.960, *p* < 0.001) and a greater tendency to utilize cognitive reappraisal (*B* = 0.800, *p* < 0.001) were associated with higher levels of growth. Conversely, a poorer workplace environment (*B* = −2.808, *p* < 0.001), greater AE severity (*B* = 2.938, *p* = 0.002), older age (*B* = −3.015, *p* = 0.006), and more years of work experience (*B* = −1.976, *p* = 0.012) were associated with lower growth. Finally, using an extroverted personality as the reference group, having an introverted personality was significantly associated with lower PTG scores (*B* = −4.057, *p* = 0.028).

**TABLE 5:** Multiple linear regression models for factors associated with PTG (*n* = 237).

Variable	Unstandardized B	Standardized *β*	*t*	*p*	Adj.R^2^	*F*	*p*
Constant	29.153	—	4.425	< 0.001	0.507	35.617	< 0.001
Cognitive reappraisal	0.800	0.326	6.236	< 0.001			
Significant others’ support	0.960	0.327	6.268	< 0.001			
Workplace environment	−2.808	−0.186	−3.956	< 0.001			
Severity of the most severe event	2.938	0.149	3.092	0.002			
Age	−3.015	−0.134	−2.768	0.006			
Length of employment	−1.976	−0.121	−2.519	0.012			
Introverted personality (vs. extroverted)	−4.057	−0.104	−2.212	0.028			

## 4. Discussion

To the best of our knowledge, this study represents the first systematic attempt to investigate PTG among healthcare professionals following involvement in a medical AE, and to identify factors associated with this growth using the post‐traumatic affective–cognitive processing model. Exposed HCPs demonstrated a mean PTG total score of 2.53 out of 5, indicating a low‐to‐moderate level of growth. Consistent with our hypotheses, support from significant others and cognitive reappraisal were significantly associated with growth alongside five other covariates.

The total PTG score of second victims exposed to medical AEs was 50.57 ± 16.33 (range: 0–100), which is lower than the pooled mean score of 66.34 (95% CI: 61.25–71.43) documented in a recent meta‐analysis of nurses exposed to various traumatic events (primarily COVID‐19 pandemic stressors) [29]. This indicates that HCPs experiencing localized medical AEs may exhibit fewer positive adaptations than those exposed to broader public health crises. Variations in trauma type have been shown to influence subsequent PTG trajectories [29], potentially because individual medical AEs generate distinct types of stress compared to systemic crises like COVID‐19. The findings offer novel evidence for the relationship between post‐traumatic stress and growth within the affective‐cognitive framework [21].

Among all dimensions, personal strength and appreciation of life scored the highest, consistent with prior studies [30, 31]. This pattern suggests that HCPs who have experienced medical malpractice often reflect positively on their clinical behavior, which can lead them to place greater value on their daily routines and professional roles. Some studies have shown that nursing staff actively restructure their cognitive perspectives after experiencing trauma [32]. Chief among these shifts is a fundamental restructuring of personal philosophy, in which individuals place greater emphasis on living in the present [33]. Confronted with frequent patient safety incidents during medical treatment, HCPs may develop a deeper appreciation for health and the meaning of life. Consequently, they come to perceive the significance of their work better, and their individual resilience is strengthened by the mission of healing the sick and saving lives [34].

This study showed that cognitive reappraisal ability was positively correlated with PTG levels, consistent with previous studies [35, 36]. The cognitive process of active reflection and re‐detection of individuals after traumatic events is an important precursor to PTG [37]. It enhances psychological resilience, promotes the positive expression of negative emotions, and helps individuals secure social support [38], thus improving their PTG. Cognitive reappraisal involves reinterpreting the emotional connotations and reconstructing core beliefs, allowing individuals to process memories constructively. Encouraging active thinking patterns helps individuals regulate their emotions and find meaning in adversity [39, 40]. Therefore, when providing psychological counseling for medical staff affected by medical AEs, clinicians should assess their emotion‐regulation abilities and support the use of cognitive reappraisal to facilitate recovery.

Perceived support from significant others, such as relatives, leaders, and colleagues, was positively correlated with PTG. Among these sources, organizational support is particularly important for second victims. In nurses exposed to AEs, discussing with a respected peer is ranked as the most valued form of support [41]. An organization’s culture and rapid response are essential to victim recovery and will ultimately affect how they move on. Providing timely and effective support at both the individual and institutional levels is positively correlated with a second victim’s recovery [10, 42]. However, in this study, support from significant others received the lowest score. This highlights the need for hospitals to actively provide organizational support and foster a supportive working environment. Implementing formal peer support networks or employee assistance programs could help alleviate psychological, physical, and occupational distress, enhance institutional belonging, reduce job insecurity, and facilitate faster recovery of clinical functioning, which are associated with better PTG.

Consistent with earlier reports [43–45], younger age and shorter length of employment were associated with higher PTG following an AE. Younger people may have greater psychological plasticity, allowing them to adapt and reshape their outlook on life more easily [43]. Regarding personality traits, introverted individuals exhibited significantly lower PTG scores than their extroverted peers, in line with Joseph’s view [21]. This might be because extroverted individuals often utilize optimistic framing, prioritize achievable goals, and more frequently employ support‐seeking, problem‐solving, and cognitive reframing strategies, which can support higher PTG [46].

Our study showed that lower AE severity was associated with higher PTG in second victims. While previous research suggests a correlation between trauma exposure and PTG, this association is rarely simply negative or positive [21]. Minor stressors may not provide sufficient stimulus to trigger cognitive restructuring, whereas overly severe trauma can overwhelm coping capacities and hinder growth [33]. In the context of medical AEs, severe outcomes such as major patient injury or death can cause deeper guilt in second victims. Furthermore, severe events often involve complex institutional reviews and administrative penalties, which can extend the accountability process and prolong self‐blame. This enduring distress can create interpersonal barriers, reduce access to social support, and limit opportunities for emotional expression, thereby impeding PTG [48]. Therefore, for second victims involved in serious AEs, hospitals should provide timely psychological counseling, avoid punitive first responses, and simplify review procedures to reduce negative emotional impact.

Several other characteristics of the sample warrant discussion. Because participants were recruited from hospital incident reporting databases, the findings reflect institutional reporting patterns.

First, nurses accounted for almost three‐quarters (71.31%) of the second victims identified, consistent with prior research [49]. However, no statistically significant difference in PTG scores was found among nurses, physicians, and technicians. Nurses have frequent, direct contact with patients during daily clinical care, which can increase their exposure to medical AEs. Additionally, this pattern indicates that nurses play an active role in AE reporting, which contributes to patient safety efforts and quality improvement initiatives. Second, the reported medical AEs were predominantly level III (no patient harm, 40.08%) or level IV (near‐miss, 38.40%), consistent with patterns observed by Xiong and Bi [50]. This indicates that HCPs across these hospitals report AEs of various severity, including those that do not cause patient harm, which supports systematic learning and suggests an open reporting culture.

This study has some limitations. First, the cross‐sectional design describes only the current levels of social support, emotional regulation, and general characteristics among second victims; it cannot track dynamic changes in PTG over time or establish clear causal relationships. Second, the sample size was limited to 237 valid responses drawn from four hospitals in Changsha, which introduces regional constraints and means the findings may not generalize nationally. Third, recall bias is a potential concern, as participants’ retrospective recollections and subjective evaluations of past events could influence their reporting of PTG. Future studies employing longitudinal designs or incorporating objective records (e.g., incident reports) could help mitigate this bias. Finally, the study focused exclusively on second victims’ PTG; incorporating evaluations of primary victims could provide additional depth in future research.

## 5. Implications for Nursing Management

Based on our empirical findings, several concrete, practical implications for nursing management emerge. First, given that support from significant others scored the lowest in this cohort, nurse managers should look beyond employees’ personal social circles and instead establish formal, institutionalized organizational support systems, such as structured peer support networks and confidential psychological counseling services. Second, because lower‐severity AEs were associated with higher levels of PTG, managers should facilitate structured, nonpunitive debriefing sessions. These sessions can guide second victims to cognitively reframe negative clinical experiences into constructive learning opportunities, thereby fostering growth without minimizing the clinical gravity of the event itself. Third, healthcare organizations should actively cultivate a culture of psychological safety. Building a supportive, nonpunitive atmosphere ensures that second victims feel safe reporting and discussing AEs, which ultimately cushions the lack of external personal support.

## 6. Conclusion

PTG among second victims of medical AEs was observed at a low to moderate level. Higher perceived support from significant others, a greater tendency to utilize cognitive reappraisal, a favorable workplace environment, lower AE severity, younger age, and shorter length of employment were significantly associated with higher PTG scores. Conversely, an introverted personality type was associated with lower growth compared to an extroverted type. These findings highlight the value of developing supportive institutional programs that encourage cognitive reappraisal strategies and provide organizational support to facilitate professional recovery and growth among HCPs.

## Funding

The study was supported by Hunan Provincial Natural Science Foundation of China (grant no. 2022JJ70161).

## Conflicts of Interest

The authors declare no conflicts of interest.

## Supporting Information

Additional supporting information can be found online in the Supporting Information section.

## Supporting information


**Supporting Information** Table 1 Assignment of independent variables in multiple linear regression analysis.

## Data Availability

Research data are not shared.
