# Supplementary material for: Navigating Recovery After Medical Adverse Events: The Role of Social Support and Cognitive Reappraisal in Second Victims’ Post‐Traumatic Growth
Source: J Nurs Manag. 2026 Jul 13;2026:5142544. doi: 10.1155/jonm/5142544 (PMC13364537; doi:10.1155/jonm/5142544)
Supplement: Supplementary file 1 — Supporting Information Table 1 Assignment of independent variables in multiple linear regression analysis. [file JONM-2026-5142544-s001.docx]

Supplementary material

***Table 1 Assignment of independent variables in multiple linear regression analysis***

| Variable | Type | Coding / Assignment | Reference group |
| --- | --- | --- | --- |
| Age* | Ordinal | ＜30 = 1, 30 ~ = 2, 40 ~ = 3, ≥50 = 4 |  |
| Marital status | Categorical (dummy) | Married = (1,0,0); Divorced = (0,1,0); Widowed = (0,0,1) | Unmarried (0,0,0) |
| Education level* | Ordinal | College = 1, Bachelor = 2, Master = 3, Doctorate = 4 |  |
| Length of employment (years)* | Ordinal | <1 = 1, 1~ = 2, 3~ = 3, 5~ = 4, ≥10 = 5 |  |
| Practice specialty | Categorical (dummy) | Physician = (1,0), Technician = (0,1) | Nurse (0,0) |
| Annual family income (10,000 RMB)* | Ordinal | <15 = 1, 15~ = 2, ≥30 = 3 |  |
| Personality type | Categorical (dummy) | Introverted = (1,0), Intermediate = (0,1) | Extroverted (0,0) |
| Workplace environment* | Ordinal | Very good = 1, Good = 2, Average = 3, Poor = 4 |  |
| Family relationship* | Ordinal | Very good = 1, Good = 2, Average = 3, Poor = 4 |  |
| Number of AEs experienced | Categorical | Once = 1, Multiple times = 2 |  |
| Severity level of medical AE* | Ordinal | Level I (warning) = 1, Level II (with harm) = 2, Level III (no harm) = 3, Level IV (near miss) = 4 |  |
| Perceived family support | Continuous | Raw score |  |
| Perceived friend support | Continuous | Raw score |  |
| Perceived support from significant others | Continuous | Raw score |  |
| Emotion regulation (cognitive reappraisal) | Continuous | Raw score |  |
| Emotion regulation (expressive suppression) | Continuous | Raw score |  |

* Entered as a continuous variable
